# Green Approach for *Rosa damascena* Mill. Petal Extract: Insights into Phytochemical Composition, Anti-Aging Potential, and Stability

**DOI:** 10.3390/antiox14050541

**Published:** 2025-04-30

**Authors:** Sawat Sopharadee, Jutinat Kittipitchakul, Nutnaree Srisawas, Waranya Neimkhum, Artit Yawootti, Thomas Rades, Wantida Chaiyana

**Affiliations:** 1 Department of Pharmaceutical Sciences, Faculty of Pharmacy, Chiang Mai University, Chiang Mai 50200, Thailand; sawat_so@cmu.ac.th (S.S.); jutinat_k@cmu.ac.th (J.K.); nutnaree_sri@cmu.ac.th (N.S.); 2 Department of Pharmaceutical Technology, Faculty of Pharmaceutical Sciences, Huachiew Chalermprakiet University, Samutprakarn 10250, Thailand; waranya.nei@hcu.ac.th; 3 Department of Electrical Engineering, Faculty of Engineering, Rajamangala University of Technology Lanna, Chiang Mai 50300, Thailand; yartit@rmutl.ac.th; 4 Department of Pharmacy, Faculty of Health and Medical Sciences, University of Copenhagen, Universitetsparken 2, 2100 Copenhagen, Denmark; thomas.rades@sund.ku.dk; 5 Center of Excellence in Pharmaceutical Nanotechnology, Faculty of Pharmacy, Chiang Mai University, Chiang Mai 50200, Thailand; 6 Multidisciplinary and Interdisciplinary School, Chiang Mai University, Chiang Mai 50200, Thailand

**Keywords:** Damask rose, green extraction, ultrasound, microwave, pulsed electric field, water, stability

## Abstract

*Rosa damascena* Mill., widely recognized for its remarkable skincare benefits, is extensively used in the cosmeceutical industry. This study introduces a novel green approach to extract bioactive compounds from *R. damascena* for cosmeceutical applications while also evaluating its stability in terms of physical, chemical, and biological properties. *R. damascena* petals were extracted using deionized water instead of organic solvents, using various green extraction methods, including infusion, microwave, ultrasound, pulsed electric field, and micellar extraction. Their chemical composition was analyzed using high-performance liquid chromatography. The extract with the highest concentration of bioactive compounds was further evaluated for its cosmeceutical properties and stability and compared with its individual chemical components. Various factors influencing stability were evaluated, including pH level (5, 7, and 9), temperature (4 °C, 30 °C, and 45 °C), and light exposure. The findings indicate that the extract obtained through microwave-assisted extraction (MAE) contained the highest concentration of bioactive constituents, with corilagin being the most abundant, followed by cyanidin-3,5-O-diglucoside, gallic acid, ellagic acid, L-ascorbic acid, and rutin, respectively. Additionally, MAE exhibited excellent antioxidant, whitening, and anti-skin-aging effects, demonstrating significantly higher activities than both the positive control (L-ascorbic acid for antioxidant effects, kojic acid for anti-tyrosinase effects, and epigallocatechin gallate and oleanolic acid for anti-skin-aging effects) and the individual chemical constituents. However, the physico-chemical and biological stability of MAE was influenced by pH, temperature, and light exposure, and as such, light-protected and controlled temperature (not exceeding 30 °C) is essential to maintain the extract’s efficacy in skincare products, and optimal formulation strategies are strongly recommended to ensure long-term stability.

## 1. Introduction

A significant demographic transition towards an aging society has been observed across numerous countries worldwide, primarily attributable to declining birth rates and increased life expectancy [1]. With the elderly population experiencing rapid growth, it becomes imperative to prioritize their healthcare and overall quality of life. One prominent aspect that undergoes inevitable changes with advancing age is the skin, which becomes increasingly vulnerable to the effects of aging. While skin aging may not directly impact physical health, the maintenance of youthful and vibrant skin holds immense potential for influencing an individual’s personality, public perception, and, subsequently, their mental well-being and overall quality of life. As a result, the prevention of skin aging before it reaches an advanced stage is encouraged at all stages of life. This prevailing societal emphasis on youthful skin has culminated in a substantial market demand for cosmetic products, particularly those offering efficacious anti-aging solutions [2].

The field of cosmeceuticals has witnessed a remarkable surge in the development of anti-aging remedies as consumers seek ways to combat the visible signs of aging and preserve a youthful appearance. The desire to maintain a youthful appearance has become a social and cultural aspiration, leading individuals of all ages to actively seek out products that can help defy the effects of time on their skin. The global market for anti-aging products has expanded rapidly, reflecting the increasing demand for innovative and scientifically proven solutions [3]. In response to this growing demand, researchers and cosmetic formulators have turned their attention to natural ingredients with potential anti-aging properties [4,5]. One such ingredient that has gained considerable attention in the cosmetic industry is *Rosa damascena* Mill., commonly known as Damask rose. This botanical species has a long history of traditional use and has been valued for its aromatic and therapeutic properties. However, its application in cosmeceuticals, particularly in anti-aging formulations, is gaining recognition due to its rich composition of bioactive compounds. *R. damascena* has been reported to contain a diverse range of bioactive compounds, including polyphenolic compounds such as quercetin, kaempferol, and ellagic acid, which possess beneficial properties for cosmetic applications [6]. Notably, these compounds exhibit antioxidant activity and tyrosinase inhibitory effects, contributing to skin whitening and providing protection against harmful ultraviolet (UV) radiation. Moreover, *R. damascena* flower extracts have demonstrated potential in reducing wrinkles and displaying strong antioxidant activities when processed into essential oils and hydrosols [7]. Infusions of *R. damascena* flowers have also exhibited a remarkable phenolic content and potent antioxidant properties [5]. Additionally, ethanolic extracts of *R. damascena* flowers have been reported to possess various biological activities, including antioxidant and anti-collagenase effects [8]. Thus, *R. damascena* flower represents an appealing natural source for anti-skin-aging ingredients.

Recently, growing concerns about environmental impact and global sustainability have been driving a green agenda that influences a wide range of human activities [9], including the integration of green chemistry principles into extraction processes. Green extraction techniques exhibit attractive characteristics, such as simplicity, versatility, high extraction efficiency, and an environmentally friendly profile [9], highlighting their importance and growing application across various industries, including the cosmetic sector. Various novel green extraction technologies have been employed for the extraction of bioactive compounds from natural resources, each offering distinct advantages and limitations. The use of green solvents, particularly water, may limit extraction efficiency; therefore, various advanced technologies have been employed to enhance extraction yield and reduce the extraction duration. High temperatures are known to enhance the solubility of many compounds, making infusion, an easy and convenient extraction method [10], more efficient. However, boiling water may compromise the stability and integrity of bioactive components in the extracts. Enhancing the extraction efficiency of water by using surfactants to form micelles can solubilize a wider range of bioactive compounds [11], but the extract may retain residual surfactants. In addition, various techniques, including microwave, ultrasound, and pulsed electric field extraction, have been widely used to disrupt plant cell membranes, resulting in more effective release of bioactive compounds and ultimately enhancing extraction efficiency [12]. Considering the advantages and limitations of various green extraction techniques, a comparative analysis is essential to identify the most efficient method for the specific material.

Therefore, the aims of the present study were to extract bioactive ingredients from *R. damascena* petals using green extraction methods and to investigate the chemical compositions and anti-aging properties of the obtained extracts. In addition, the physical and chemical stability of the *R. damascena* petal extracts obtained using green extraction methods were also evaluated. The results of this study will provide valuable insights into the cosmeceutical potential of *R. damascena* petal extracts, contributing to the development of effective and sustainable anti-aging products in the cosmetic industry.

## 2. Materials and Methods

### 2.1. R. damascena Petals

Fresh flowers of *R. damascena* were obtained as a gift from an organic farm owned by Magicus Health & Beauty Care Co., Ltd., located in Mae Taeng District, Chiang Mai, Thailand. The diagnostic pharmacognostic characters of the plant sample were identified and authenticated by Ms. Wannaree Charoensup, a botanist at the Herbarium, Department of Pharmaceutical Sciences, Faculty of Pharmacy, Chiang Mai University. The specimen, numbered 0023313, was kept at the Herbarium of the Faculty of Pharmacy, Chiang Mai University. Following careful dissection of the *R. damascena* flower, the petals were separated and subjected to gentle drying at 40 °C in a hot air oven (UN110, Memmert GmbH + Co. KG, Schwabach, Germany). Subsequently, the dried *R. damascena* petals were ground into a fine powder using a Moulinex blender (Moulinex, Paris, France) and kept in a hermetically sealed aluminum bag until their application in subsequent investigations.

### 2.2. Chemicals

Bovine testicular hyaluronidase (E.C.3.2.1.3.5), metalloproteinase-1 (MMP-1) from *Clostridium histolyticum* (ChC-E.C.3.4.23.3), synthetic peptide 2-furanacryloyl-Leu-Gly-Pro-Ala (FALGPA), N-Succinyl-Ala-Ala-Ala-p-nitroanilide (AAAVPN), tyrosinase from mushroom (EC 1.14.18.1), L-ascorbic acid, kojic acid, gallic acid, cyanidin-3,5-O-diglucoside, corilagin, rutin, ellagic acid, quercetin, epigallocatechin gallate (EGCG), oleanolic acid, 2,2-Diphenyl-1picrylhydrazyl (DPPH), 2,4,6 tripyridyl-s-triazine (TPTZ), aluminum chloride (AlCl_3_), 2,2-azino-bis3-ethylbenzothiazoline-6-sulfonic acid (ABTS), ferric chloride (FeCl_3_), ferrous chloride (FeCl_2_), ferrous sulfate (FeSO_4_), Folin–Ciocalteu reagent, 6-hydroxy-2,5,7,8-tetramethylchroman-2-carboxylic acid (Trolox), hyaluronic acid, hydrochloric acid (HCl), formic acid (HCOOH), sodium acetate (CH_3_COONa), potassium acetate (CH_3_COOK), potassium persulphate (K_2_S_2_O_8_), sodium acetate (C_2_H_3_NaO_2_), sodium carbonate (Na_2_CO_3_), sodium chloride (NaCl), Tricine base, sodium dihydrogen phosphate (NaH_2_PO_4_), disodium phosphate (Na_2_HPO_4_), and sodium phosphate (Na_3_PO_4_) were purchased from Sigma-Aldrich (St. Louis, MO, USA) and all of analytical grade. Bovine serum albumin (BSA) (catalog number: B14) was purchased from Gibco™ (Thermo Fisher Scientific, Waltham, MA, USA). Polysorbate 20 (Tween^®^ 20) and Polysorbate 80 (Tween^®^ 80) were purchased from Namsiang Co., Ltd. (Bangkok, Thailand). Acetonitrile was high-performance liquid chromatography (HPLC) grade and purchased from Labscan, Ltd. (Dublin, Ireland). Deionized (DI) water was purchased from RCI Labscan Co., Ltd. (Bangkok, Thailand).

### 2.3. Extraction of R. damascena Petals

**Figure 1:**
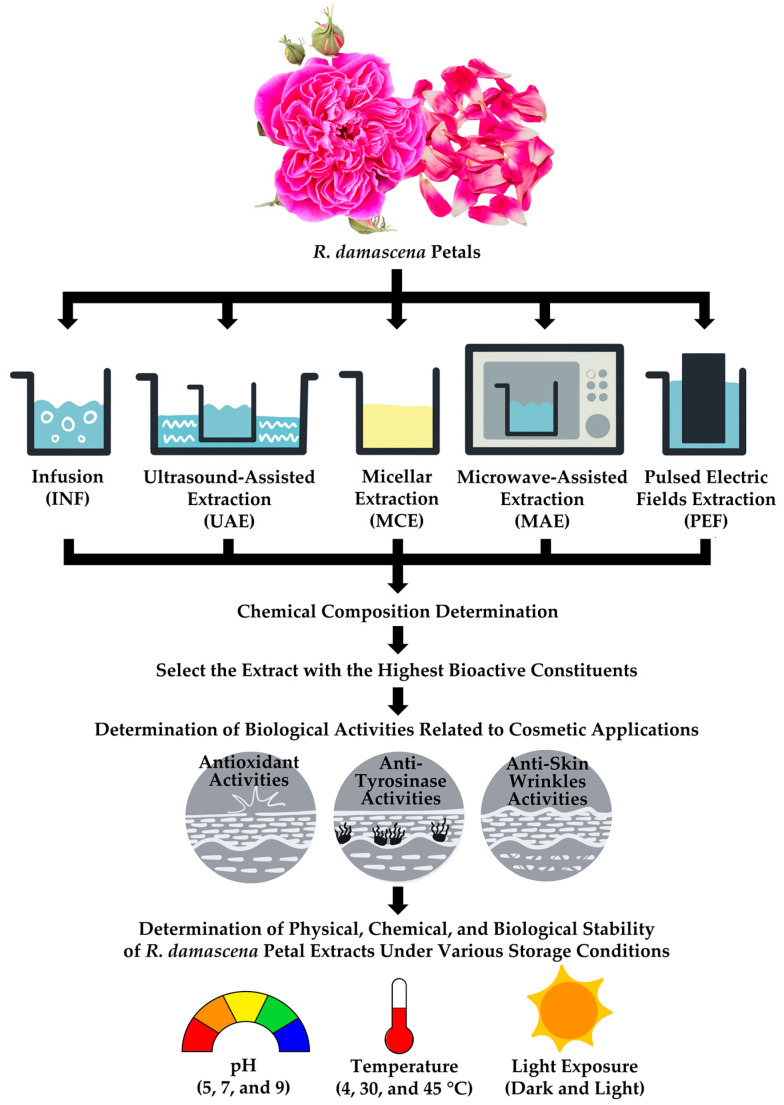
A schematic representation of green extraction techniques applied to *R. damascena* petal powder, along with an overview of the methodology.

The dried powder of *R*. *damascena* petals was extracted using various green extraction methods to compare their efficiency in yielding bioactive compounds, and an overview of the subsequent methodology is presented in Figure 1.

#### 2.3.1. Infusion (INF)

The dried powder of *R. damascena* petals was infused in boiling DI water at a weight ratio of 1:25 for 10 min with continuous stirring. After vacuum-assisted filtration using Whatman No. 1 filter paper (Merck KGaA, Darmstadt, Germany) and a vacuum pump (TC-501V, Worldwide Trade Thai Co., Ltd., Bangkok, Thailand), the filtrate was collected and pre-cooled to −20 °C on the freeze-drying tray with a diameter of 30 cm. The DI water that was used as a solvent was then removed by freeze-drying (FreeZone 4.5, Labconco, KS, USA). Prior to initiating the freeze-drying process, the condenser of the freeze dryer was pre-cooled to −40 °C for 30 min. Subsequently, the tray containing the frozen samples was placed in the drying chamber. The vacuum pump was then turned on until the pressure reached 0.12 mbar. After a drying process of 72 h, the vacuum was slowly released until the pressure was stabilized. The dried samples were then removed and stored at 4 °C until further investigation. Infusion extracts were obtained and stored at 4 °C until further investigations.

#### 2.3.2. Ultrasound-Assisted Extraction (UAE)

The dried powder of *R. damascena* petals was extracted with DI water at a weight ratio of 1:25 with the assistance of an ultrasound, at room temperature, for 10 min using an ultrasound bath (Elmasonic S30H, Elma Schmidbauer GmbH, Singen, Germany) with an ultrasonic frequency of 37 kHz, a power level of 80 W, and a maximum volume capacity of 2.75 L. After filtration, the filtrate was collected and subjected to freeze-drying and storage, as described in Section 2.3.1.

#### 2.3.3. Micellar Extraction (MCE)

The dried powder of *R. damascena* petals was extracted using micellar solutions of Tween^®^ 20 or Tween^®^ 80 at concentrations of 3% *w*/*w* and at a weight ratio of 1:25 for 10 min under constant stirring. This was followed by filtration, freeze-drying, and storage, as outlined in Section 2.3.1.

#### 2.3.4. Microwave-Assisted Extraction (MAE)

The dried powder of *R. damascena* petals was extracted using DI water at a weight ratio of 1:25 with the assistance of microwave irradiation for 3 min using a microwave oven (ER-SM20(W)TH, Toshiba Thailand Co., Ltd., Bangkok, Thailand) with an operating voltage of 220 V/50 Hz, a power level of 800 W, and a maximum volume capacity of 20.0 L. This was followed by filtration, freeze-drying, and storage, as outlined in Section 2.3.1.

#### 2.3.5. Pulsed Electric Field Extraction (PEF)

The dried powder of *R. damascena* petals was extracted using DI water at a weight ratio of 1:25 using a PEF set at 15 kV/cm for 10 min. The PEF, developed by Dr. Artit Yawootti (Department of Electrical Engineering, Faculty of Engineering, Rajamangala University of Technology Lanna, Chiang Mai, Thailand), was operated using a voltage of 220 V/50 Hz, a power level of 500 W, and a maximum volume capacity of 0.57 L. The system utilized rotating gap switches to generate a high-voltage electric field of 15 kV/cm directed into the sample treatment chamber. The pulse duration and pulse repetition frequency were kept constant at 1 μs and 10 Hz, respectively. This was followed by filtration, freeze-drying, and storage, as outlined in Section 2.3.1.

### 2.4. Chemical Composition Determination of R. damascena Petal Extracts

The contents of ascorbic acid (1), gallic acid (2), cyanidin-3,5-O-diglucoside (3), corilagin (4), rutin (5), ellagic acid (6), and quercetin (7) of different extracts from *R. damascena* petals were determined using HPLC (Hewlett-Packard, Palo Alto, CA, USA) with a reversed phase column (Eurospher II 100-5 C18, 250 × 4.6 mm, 5 µm, Knauer, Berlin, Germany) connected with a precolumn (Eurospher II 100-5 C18, 10 mm × 4.6 mm, 5 µm, Knauer, Berlin, Germany). The mobile phase containing (A) 0.05% formic acid in acetonitrile and (B) 0.05% formic acid in deionized water was used in gradient elution as follows: 0–8 min, 10–20% of A; 8–28 min, 20–30% of A; 28–30 min, 20–10% of A; and 30–35 min, 10% of A. A flow rate of 1 mL/min was used at ambient temperature. The sample injection volume was 10 µL, and the UV detector was set at a wavelength of 280 nm. Each sample was filtered through a 0.45 µm nylon filter membrane prior to the injection. A calibration curve of each standard compound was constructed and used for quantitative determination. All experiments were conducted independently in triplicate.

### 2.5. Determination of Biological Activities Related to Cosmetic Applications

#### 2.5.1. Antioxidant Activities

- 2,2-Diphenyl-1-Picrylhydrazyl (DPPH) Assay

The DPPH^•^ radical scavenging activity of *R. damascena* petal extract and its chemical constituents were assessed using a DPPH assay based on the method by Brem et al. (2004) [13] with some modifications [7]. In this assay, 20 µL of the sample solution of MAE or its chemical constituents was mixed with 180 µL of a DPPH^•^ solution, prepared by dissolving 167 mM DPPH in methanol. The mixture was incubated in the dark at room temperature for 30 min, and absorbance was measured at 520 nm using a multimode detector (SPECTROstar Nano, BMG Labtech, Offenburg, Germany). The percentage of DPPH^•^ radical scavenging was calculated with the following equation:

DPPH^•^ radical scavenging (%) = [(a − b)/a] × 100, (1)

where a and b represent the absorbance of the mixture without and with the presence of the samples, respectively. Various concentrations of the extracts, ranging from 0.0625 to 1.00 mg/mL, were evaluated, and their DPPH^•^ radical scavenging activities were plotted against the logarithmic concentrations to create dose–response curves. IC_50_ values were calculated from these curves using GraphPad Prism (version 8.0.2, GraphPad Software, Boston, MA, USA). L-ascorbic acid was employed as a positive control. All experiments were conducted independently in triplicate.

- 2,2’-Azino-Bis(3-Ethylbenzothiazoline-6-Sulfonic Acid) (ABTS) Assay

The ABTS^•+^ radical scavenging activity of *R. damascena* petal extract and its chemical constituents were assessed using an ABTS assay based on the method by Miller and Rice-Evans (1996) [14] with some modifications [7]. In this assay, a stock solution of ABTS^•+^ radicals was prepared by combining a 7 µM aqueous solution of ABTS with a 2.45 mM K_2_S_2_O_8_ aqueous solution, and the mixture was incubated in the dark at room temperature for 16 h. Prior to the experiment, the ABTS^•+^ radicals stock solution was diluted with DI water at a volume ratio of 1:20. Subsequently, 20 µL of the sample solution of MAE or its chemical constituents was mixed with 180 µL of an ABTS^•+^ solution and incubated for 5 min at room temperature. The absorbance of mixtures containing *R. damascena* petal extracts at a final concentration of 0.1 mg/mL was measured at 750 nm using a multimode detector (SPECTROstar Nano, BMG Labtech, Offenburg, Germany). The results were expressed as Trolox equivalent antioxidant capacity (TEAC) values, calculated using the following equation:

TEAC (µg Trolox/mg extract) = (A – 0.5910)/0.0375, (R^2^ = 0.9347), (2)

where A is the absorbance of the sample. L-ascorbic acid was employed as a positive control. All experiments were conducted independently in triplicate.

- Ferric Reducing Antioxidant Power (FRAP) Assay

The ferric reducing antioxidant power of *R. damascena* petal extract and its chemical constituents was assessed using a FRAP assay based on the method by Saeio et al. (2011) [15] with some modifications [7]. In this assay, the FRAP solution was freshly prepared by combining 0.3 M acetate buffer at pH 3.6, 10 mM TPTZ in 40 mM HCl, and 20 mM ferric chloride solution. Subsequently, 20 µL of the sample solution of MAE or its chemical constituents was mixed with 180 µL of the FRAP solution and incubated at room temperature for 5 min. Absorbance was measured at 595 nm using a multimode detector (SPECTROstar Nano, BMG Labtech, Offenburg, Germany). The results were reported in terms of equivalent capacity (EC_1_), which represented ferric reducing ability equivalent to 1 µM FeSO_4_, calculated using the following equation:

EC_1_ (µM FeSO_4_/mg extract) = (A – 1.2764)/0.2132, (R^2^ = 0.9909), (3)

where A is the absorbance of the sample. L-ascorbic acid was employed as a positive control. All experiments were conducted independently in triplicate.

#### 2.5.2. Anti-Tyrosinase Activities

The anti-tyrosinase activities of *R. damascena* petal extract and its chemical constituents were assessed using a modified spectrophotometric assay based on the method by Saeio et al. (2011) [15] with some modifications [7]. In this assay, the tyrosinase solution was prepared at a concentration of 250 units/mL from mushroom tyrosinase in 20 mM of phosphate buffer at pH 6.8. Subsequently, 10 µL of the sample solution of MAE or its chemical constituents was mixed with 30 µL of the tyrosinase solution and incubated at room temperature for 10 min. Following the addition of 100 µL 12 mM L-3,4-dihydroxyphenylalanine (L-DOPA), the resulting mixture was further incubated for 30 min. Absorbance was measured at 595 nm using a multimode detector (SPECTROstar Nano, BMG Labtech, Offenburg, Germany). The percentage of tyrosinase inhibition was calculated with the following equation:

Tyrosinase inhibition (%) = [(a − b)/a] × 100,(4)

where a and b represent the absorbance of the mixture without and with the presence of the samples, respectively. Kojic acid was employed as a positive control. All experiments were conducted in triplicate.

#### 2.5.3. Anti-Skin Wrinkles Activities

- Collagenase Inhibitory Activities

The anti-collagenase activities of *R. damascena* petal extract and its chemical constituents were assessed using a modified spectrophotometric enzyme substrate assay based on the method by Thring et al. (2009) [16] with some modifications [17]. In this assay, 50 mM of Tricine buffer at pH 7.5 containing 400 mM NaCl and 10 mM CaCl_2_ was used as the solvent for the enzymatic reaction. In brief, 0.5 units/mL of collagenase from *C. histolyticum* was mixed with the sample solution of MAE or its chemical constituents and the solvent at the volume ratio of 1:1:4 and incubated for 15 min. Subsequently, 2.0 M FALGPA was added, and absorbance at 340 nm was immediately measured in kinetic mode over 20 min using a multimode detector (SPECTROstar Nano, BMG Labtech, Offenburg, Germany). Collagenase inhibition was calculated using the following equation:

Collagenase inhibition (%) = [(a − b)/a] × 100,(5)

where a and b represent the reaction rate of the system without and with the presence of the sample, respectively. Oleanolic acid and EGCG were employed as positive controls. All experiments were conducted independently in triplicate.

- Elastase Inhibitory Activities

The anti-elastase activities of *R. damascena* petal extract and its chemical constituents were assessed using a modified spectrophotometric enzyme substrate assay based on the method by Thring et al. (2009) [16] with some modifications [18]. In this assay, 0.2 M of Tris-HCl buffer at pH 8.0 was used as the solvent for the enzymatic reaction. In brief, 0.03 units/mL of elastase from porcine pancreas was mixed with the sample solution of MAE or its chemical constituents at the volume ratio of 4:1 and incubated for 15 min. Subsequently, 1.6 mM AAAVPN was added, and absorbance at 410 nm was immediately measured in kinetic mode over 20 min using a multimode detector (SPECTROstar Nano, BMG Labtech, Offenburg, Germany). Elastase inhibition was calculated using the following equation:

Elastase inhibition (%) = [(a − b)/a] × 100,(6)

where a and b represent the reaction rate of the system without and with the presence of the sample, respectively. Oleanolic acid and EGCG were employed as positive controls. All experiments were conducted in triplicate.

- Hyaluronidase Inhibitory Activities

The anti-hyaluronidase activities of *R. damascena* petal extract and its chemical constituents were assessed using a modified spectrophotometric enzyme substrate assay based on the method by Chaiyana et al. (2019) [19] with some modifications [18]. In this assay, phosphate buffer at pH 5.3 was used as the solvent for the enzymatic reaction. In brief, 1.5 units/mL hyaluronidase from bovine testis was mixed with the sample solution of MAE or its chemical constituents at the volume ratio of 5:1 and incubated for 10 min. Subsequently, 0.03% *w*/*v* hyaluronic acid was added and incubated at 37 °C for an additional 45 min. Following incubation, an acid bovine serum albumin solution consisting of acetic acid, sodium acetate, and bovine serum albumin was added. After a further 10 min of incubation, the absorbance of the mixture was measured at 600 nm using a multimode detector (SPECTROstar Nano, BMG Labtech, Offenburg, Germany). Hyaluronidase inhibition was calculated using the following equation:

Hyaluronidase inhibition (%) = [(a − b)/a] × 100,(7)

where a and b represent the absorbance of the mixture without and with the presence of the sample, respectively. Oleanolic acid and EGCG were employed as positive controls. All experiments were conducted in triplicate.

### 2.6. Determination of Physical, Chemical, and Biological Stability of R. damascena Petal Extracts

#### 2.6.1. Storage Conditions

The *R. damascena* petal extract was kept under various storage conditions, including at different pH levels (5, 7, and 9), temperatures (4, 30, and 45 °C), and exposure to light or light-protected environments, over a period of 24 h. The extracts were subsequently evaluated for their physical, chemical, and biological stability.

#### 2.6.2. Physical Stability Evaluation

After storage in various conditions, the physical characteristics of the *R. damascena* extract were examined in terms of physical appearance by visual inspection, and the color of each extract was evaluated using a colorimeter (Cortex Technology Aps, Plastvaeget 9, Hadsund, Denmark). The L*, a*, and b* values, which represent the color characteristics of the *R. damascena* extract, were recorded. The L* value (lightness) indicates the brightness level of the sample, ranging from 0 (black) to 100 (white). The a* value represents the green-red color component, with positive values indicating a shift towards red and negative values indicating a shift towards green. The b* value reflects the blue-yellow color component, with positive values indicating a shift towards yellow and negative values indicating a shift towards blue. The evaluations were conducted at the following time intervals: 0, 5, 10, 15, and 30 min and 1, 2, 4, 6, 8, and 24 h. All experiments were performed independently in triplicate.

#### 2.6.3. Chemical Stability Evaluation

After storage under various conditions for 24 h, the chemical characteristics of the *R. damascena* petal extracts were analyzed using HPLC, as previously described. The remaining content of each individual compound was calculated using the following equation:

Remaining content (%) = (a/b) × 100,(8)

where a represents the content of the individual compound in the *R. damascena* petal extract after storage under various conditions for 24 h, and b represents the content of the individual compound prior to the stability test. All experiments were performed independently in triplicate.

#### 2.6.4. Biological Stability Evaluation

After storage under various conditions for 24 h, the biological activities of the *R. damascena* petal extracts were examined in terms of antioxidant, anti-tyrosinase, and anti-skin wrinkle activities, as mentioned above. The remaining biological activities of each *R. damascena* petal extract were calculated using the following equation:

Remaining activities (%) = (a/b) × 100,(9)

where a represents the biological activities of the individual compound in the *R. damascena* petal extract after storage under various conditions for 24 h, and b represents the biological activities of the individual compound prior to the stability test. All experiments were performed independently in triplicate.

### 2.7. Statistical Analysis

The data are presented as means, and S.D. GraphPad Prism (version 8.0, GraphPad Software) was used to perform a paired sample *t*-test and one-way analysis of variance (ANOVA). Then, a post hoc test was performed to determine statistical significance. The level for statistical significance was established at *p* < 0.05.

## 3. Results and Discussion

### 3.1. R. damascena Petal Extracts

**Figure 2:**
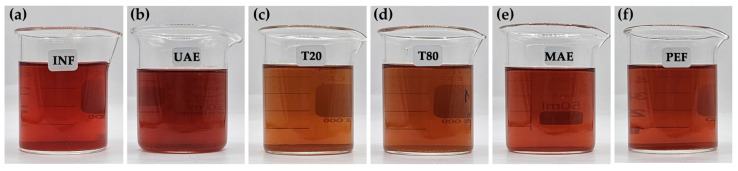
The external appearance of *R. damascena* petal extracted from DI water using various green extraction methods: (**a**) infusion (INF); (**b**) ultrasonic-assisted extraction (UAE); (**c**) micellar extraction (MCE) using Tween^®^ 20; (**d**) micellar extraction (MCE) using Tween^®^ 80; (**e**) microwave-assisted extraction (MAE); and (**f**) pulsed electric field extraction (PEF).

The *R. damascena* petal extracts obtained through various green extraction methods, including INF, UAE, MCE, MAE, and PEF, exhibited red-brown solutions, each with a distinct hue, as shown in Figure 2. The extracts from MCE using both Tween^®^ 20 and Tween^®^ 80 were observed to have a slightly orange hue, attributed to the yellowish color of the surfactants.

### 3.2. Chemical Compositions of R. damascena Petal Extracts

**Figure 3:**
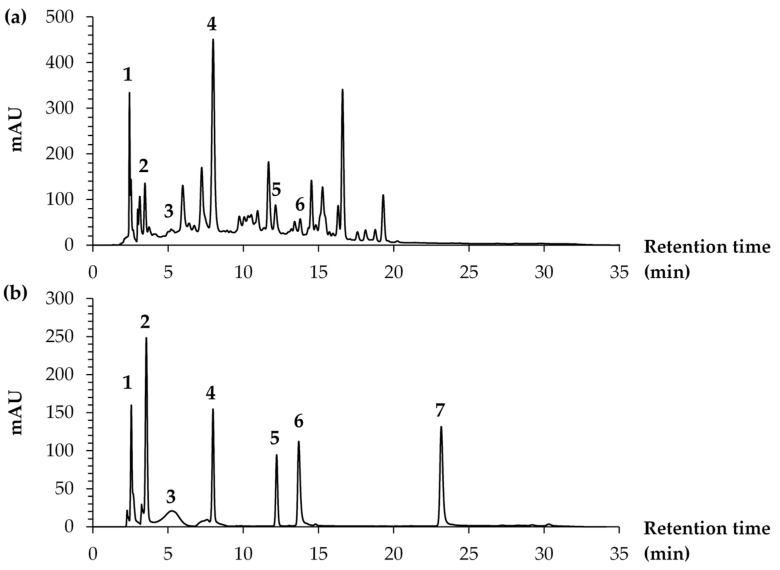
HPLC chromatograms of (**a**) *R. damascena* extract obtained using microwave extraction (MAE) for 3 min and (**b**) standard compounds: (1) L-ascorbic acid; (2) gallic acid; (3) cyanidin-3,5-O-diglucoside; (4) corilagin; (5) rutin; (6) ellagic acid; and (7) quercetin.

The HPLC chromatograms of the *R. damascena* petal extract (MAE) and standard compounds, including L-ascorbic acid (1), kojic acid (2), gallic acid (3), cyanidin-3,5-O-diglucoside (4), corilagin (5), rutin (6), ellagic acid (7), and quercetin (8), are shown in Figure 3. The petal extracts of *R. damascena* were found to contain a range of chemical constituents, as demonstrated by the multiple peaks observed in the HPLC chromatogram. The retention times of each standard compound, along with their corresponding amount in the *R. damascena* petal extracts obtained using the green extraction method, are presented in Table 1. It is essential to note that the quantity of each compound did not directly correlate with the HPLC peak height or area under the curve (AUC) analyzed at a fixed wavelength of 280 nm but was instead derived from calculations based on the standard curve, with variations in maximum absorbance leading to differing slopes and distinct formulations for quantitative analysis.

In the current study, the MAE emerged as the most effective method, yielding an extract with the highest concentrations of all compounds, with corilagin identified as the most prominent, followed by cyanidin-3,5-O-diglucoside, gallic acid, ellagic acid, and small amounts of L-ascorbic acid and rutin. While numerous studies have reported on the chemical composition of *R. damascena*, most have focused primarily on essential oils or alcoholic extracts. The phytochemical profile of the aqueous extract has been less frequently explored in the existing literature. Nevertheless, rose petals are known to contain a variety of bioactive compounds, including phenolic acids, flavonoids, flavanols, anthocyanins, hydrolysable tannins, and L-ascorbic acid [20,21]. A broad range of phenolic compounds, including several types of anthocyanins and more than thirty flavonols, has been reported, with compositional profiles varying significantly across species and cultivars [22]. The chemical composition of the *R. damascena* petal extract observed in the present study aligned with previous studies that highlighted the richness of the Rosaceae family in corilagin, a naturally occurring water-soluble retrogallic acid tannin [23], as well as phenolic compounds such as cyanidin-3,5-di-O-glucoside, gallic acid, and ellagic acid [24,25,26]. Through the application of MAE, a significant enhancement in the corilagin content was observed, along with substantial increases in other bioactive compounds, compared to other green extraction methods. Notably, gallic acid was present in approximately ten times higher amounts in MAE, cyanidin-3,5-di-O-glucoside was found in five times higher amounts, and the amounts of L-ascorbic acid, gallic acid, and ellagic acid were roughly doubled compared to the other methods. In contrast, the rutin content was present in only a modestly higher amount. A likely explanation is that the microwave treatment caused a disruption in the plant tissues, resulting in the rapid release of chemical substances from the cells into the surrounding extractant during the disruption process [27,28]. Additionally, microwave is a form of electromagnetic radiation that propagates as waves, which can be absorbed when passing through a medium and converted into thermal energy, leading to increased solubility of the extracting compounds and consequently enhancing the extraction efficiency [29]. In general, the choice of solvent takes into account both its affinity for the target compound and its ability to absorb microwave energy, as polar molecules like water efficiently absorb energy, causing rapid internal heating that may lead to vaporization and the rupture of cell walls and plasma membranes [30].

On the other hand, MCE using both Tween^®^ 20 and Tween^®^ 80 yielded different results, with ellagic acid being the most abundant compound detected rather than corilagin. The likely explanation is the poor water solubility of ellagic acid (9.7 ± 3.2 μg/mL) [31], which leads to inefficient extraction by water but more effective extraction by MCE, where surfactants act as solubilizers. Therefore, the MCE approach is recommended as effective and suitable for extracting compounds with low water solubility. However, given that MAE yielded the highest concentration of bioactive compounds among the various green extraction methods, the extract obtained through this technique was selected for subsequent biological activity investigations. Rather than a comprehensive comparison of all green extraction techniques, concentrating on the bioactivities of the MAE, and conducting a thorough analysis of the key active constituents, is likely to yield more significant and valuable insights.

### 3.3. Antioxidant Activities of R. damascena Petal Extracts

MAE, which produced the *R. damascena* petal extract with the highest concentration of bioactive compounds, was assessed for its antioxidant activities and compared with its individual bioactive constituents, including L-ascorbic acid, gallic acid, cyanidin-3,5-O-diglucoside, corilagin, rutin, and ellagic acid, as detailed in Table 2. MAE was found to exhibit the significantly highest antioxidant activities through both radical scavenging and reducing ability (*p* < 0.05). Surprisingly, the antioxidant activities observed using MAE were found to be significantly superior to those of the pure standard compounds, despite these compounds being its chemical constituents. Corilagin, identified as its major component, was not responsible for the observed antioxidant activities as it exhibited significantly lower activities compared to the *R. damascena* petal extract (*p* < 0.05). Among the various individual components, gallic acid emerged as the most potent DPPH^•^ radical scavenger, while rutin exhibited the strongest scavenging activity against ABTS^•+^ radicals. On the other hand, L-ascorbic acid, gallic acid, and ellagic acid demonstrated comparable potency in ferric reducing ability. This suggested that the synergistic interactions within the MAE may contribute to enhanced antioxidant efficacy beyond the individual effects of the isolated compounds.

The previous study emphasized the distinct potential of *R. damascena* petal extract, positioning it as superior to other herbal extracts [7]. Additionally, the current findings reveal even stronger results, demonstrating that the antioxidant activities of the extract surpassed those of the individual pure components, further confirming its unique effectiveness. The key factor for *R. damascena* petal extract to exhibit exceptionally strong antioxidant activities is its rich variety of bioactive components, including L-ascorbic acid, phenolic acids, flavonoids, anthocyanins, ellagitannins, and other phenolic compounds. Although the contents of L-ascorbic acid (9.97 ± 0.01 mg/g extract) and gallic acid (72.56 ± 0.03 mg/g extract) were not as high as corilagin (213.59 ± 0.01 mg/g extract) when using MAE, they are likely the main bioactive compounds responsible for the antioxidant activity due to their potent radical scavenging and ferric reducing properties, as shown in Table 2. L-ascorbic acid, a well-known strong antioxidant, primarily acts as a donor of single hydrogen atoms, with its radical anion monodehydroascorbate preferentially reacting with radicals over non-radical compounds [32] while als playing a key role in regulating iron uptake by reducing ferric Fe^3+^ to ferrous Fe^2+^ ions [33]. In addition, gallic acid, a secondary metabolite widely found in the plant kingdom, functions as a natural antioxidant through its radical scavenging capacity, which is attributed to the availability of hydroxyl groups and the potential for stabilizing the resulting phenolic radicals via hydrogen bonding or extended electron delocalization [34]. Additionally, gallic acid is recognized for its strong reducing power [35].

As ellagic acid is a dimeric derivative of gallic acid, a similar trend in antioxidant activities was observed, with pronounced DPPH^•^ scavenging activity and ferric reducing capacity but limited ABTS^•+^ radical scavenging activity. However, the lower activity of ellagic acid compared to gallic acid can be attributed to its steric structure, which may restrict the accessibility of its hydroxyl groups, thereby limiting its potential for antioxidant action.

On the other hand, anthocyanins, a class of flavonoids generally known to be responsible for the color of rose petals [36], are not as potent in antioxidant activity. Among various anthocyanins, cyanidin 3,5-diglucoside is most commonly detected in the petals of a wide range of rose varieties [37]. Although cyanidin-3-O-glucoside is sometimes predominant, cyanidin-3,5-O-diglucoside has been reported to remain significantly more stable [38,39]. Previous studies have suggested a strong, statistically significant positive correlation between the total anthocyanin content and antioxidant activity in rose petals, with red-colored petals being associated with higher antioxidant activity [40]. However, comparisons among individual anthocyanins, L-ascorbic acid, and other phenolic compounds in terms of antioxidant activities are rarely reported. The current study revealed that cyanidin-3,5-O-diglucoside was much less potent than MAE, as well as L-ascorbic acid and other phenolic compounds, in both radical scavenging and ferric reducing capacity. However, anthocyanins are commonly used as a marker for rose petal analysis due to their consistent association with petal color, which is visually observable and serves as a key indicator for quality control [41].

Corilagin, the primary compound in MAE, demonstrated potent ABTS^•+^ scavenging activity, comparable to that of L-ascorbic acid. In contrast, it exhibited weaker DPPH^•^ scavenging activity and ferric reducing capacity compared to the other compounds. The uncorrelated radical scavenging activities of corilagin against both ABTS^•+^ and DPPH^•^ were evident. A likely explanation could be due to different types of ABTS^•+^ and DPPH^•^ radicals. DPPH^•^ generates a relatively stable nitrogen-centered free radical, while ABTS^•+^ forms a more stable, sulfur-centered radical cation [42]. A hydrogen atom donor that reduces the ABTS^•+^ cation to its non-radical form would give positive results in the ABTS assay, while free radical scavengers or hydrogen donors can give positive results in the DPPH assay [42]. In addition, DPPH^•^ is generally more sensitive to non-polar or lipophilic antioxidants, whereas ABTS^•+^ is often more sensitive to hydrophilic antioxidants as the ABTS^•+^ radical can dissolve in both polar and non-polar solvents. Corilagin, a water-soluble polyphenolic tannin, is therefore likely to exhibit greater scavenging activity in the ABTS assay than in the DPPH assay.

Rutin, a flavonol glycoside commonly found in various plants and presented in trace amounts in the *R. damascena* petal extract in this study, demonstrated predominant ABTS^•+^ radical scavenging activity but exhibited reduced potency in DPPH^•^ radical scavenging activity and ferric reducing ability. It was observed that the ABTS^•+^ radical scavenging activity of rutin was significantly higher than that of L-ascorbic acid, whereas its DPPH^•^ radical scavenging and ferric reducing abilities were approximately one-tenth and one-half, respectively. The results align with those of a study by Gęgotek et al. (2019), which found that rutin exhibited similar ABTS^•+^ radical scavenging activity to L-ascorbic acid, while L-ascorbic acid showed three times greater antioxidant activity than rutin in the FRAP assay [43]. The very low potency of rutin in DPPH^•^ inhibition has also been reported by Kavimani et al. (2014), who found an IC_50_ value of 0.62 ± 0.04 µg/mL for ABTS^•+^ radical scavenging activity but noted that it was ten times less potent against DPPH^•^ radical scavenging activity, with an IC_50_ value of 6.15 ± 0.13 µg/mL [44]. Although rutin was not potent across all antioxidant mechanisms tested in the current study, its presence had a synergistic effect or enhanced the reduction ability of ascorbic acid [43], resulting in the potent antioxidant activities of the *R. damascena* petal extract being higher than that of L-ascorbic acid alone. This could likely explain why MAE exhibited the most potent antioxidant activities compared to all the pure standard compounds.

Oxidative damage is widely recognized as a key factor in aging and various degenerative diseases [45]. The *R. damascena* petal extract, which contains a range of natural antioxidants, has shown the ability to neutralize free radicals through several mechanisms. Additionally, the possible additional or synergistic effects of these antioxidants may further enhance their individual activity, making the extract a promising candidate for use as an antioxidative agent. This suggests that MAE could offer significant protective benefits against conditions related to oxidative stress, including skin aging.

### 3.4. Anti-Trosinase Activities of R. damascena Petal Extracts

The anti-tyrosinase activity of the *R. damascena* petal extract and its individual bioactive constituents is presented in Table 3. Tyrosinase catalyzes the conversion of L-tyrosine into L-DOPA and DOPA quinone, leading to melanin production, making enzyme inhibition a key strategy for skin whitening [46]. The findings from this research reveal that *R. damascena* extract demonstrated anti-tyrosinase activity similar to that of kojic acid, a commonly used skin-lightening agent in the cosmetic industry. However, kojic acid is associated with certain drawbacks, such as the potential to cause contact dermatitis, photo-damage, and possible mutagenic effects on the skin [47,48]. The strong anti-tyrosinase activity of *R. damascena* extract, attributed to the synergistic interactions of its diverse bioactive compounds, positions MAE as a natural whitening agent and a promising alternative to kojic acid derived from fungal fermentation [49]. Although different substrates were used, all individual bioactive compounds from *R. damascena* extract exhibited similar potential in inhibiting the tyrosinase enzyme when the substrates were tyrosine and L-DOPA, with L-ascorbic acid showing the highest potency, followed by cyanidin-3,5-O-diglucoside, gallic acid, corilagin, ellagic acid, and rutin, respectively. However, the potential of L-ascorbic acid was much less than that of the *R. damascena* extract, suggesting a synergistic effect from the combination of bioactive compounds present in the extract. The results are consistent with those of the previous study, highlighting that the tyrosinase inhibitory effect of *R. damascena* ethanolic extract was greater than that of L-ascorbic acid [50].

### 3.5. Anti-Aging Activities of R. damascena Petal Extracts

Since human fibroblast collagenase (MMP-1) is the most ubiquitously expressed interstitial collagenase and plays a prominent role in the initial cleavage of the extracellular matrix (ECM) [51], an MMP-1 inhibitor could serve as an anti-skin-aging agent by retarding extracellular matrix degradation. Elastase is a member of the chymotrypsin family of proteases and is primarily responsible for the breakdown of elastin, as well as collagen, fibronectin, and other ECM proteins [52]. Therefore, elastase inhibitors have the potential to be effective cosmetic ingredients to combat skin aging due to their role in preventing the loss of skin elasticity [53]. On the other hand, the upregulation of hyaluronidase due to photo-exposure is associated with increased levels of fragmented hyaluronic acid [54]. Among the ECM components, glycosaminoglycans, especially hyaluronic acid, are of high importance as they provide structure and viscosity to the ECM, regulate cell proliferation and locomotion, and retain a significant amount of water molecules in the inter-fibrillar spaces [54]. A hyaluronidase inhibitor is therefore essential for anti-skin aging by preventing the breakdown of hyaluronic acid and preserving skin hydration and elasticity.

An overview of the anti-aging activities of the *R. damascena* petal extract and its individual bioactive constituents, in terms of their inhibitory effects against collagenase, elastase, and hyaluronidase, is presented in Table 4. The findings from this study identify *R. damascena* extract as a highly effective anti-aging ingredient, demonstrating strong inhibitory effects against enzymes involved in the degradation of the extracellular matrix in the human skin, including collagenase, elastase, and hyaluronidase. While both positive controls exhibited comparable effectiveness in inhibiting collagenase to that of *R. damascena* extract, the extract was found to be more effective in inhibiting elastase and superior in inhibiting hyaluronidase compared to both EGCG and oleanolic acid. The chemical constituent primarily responsible for the anti-collagenase activity of *R. damascena* extract appears to be gallic acid, given its strong biological effects. The findings align with a previous study that proposed that gallic acid inhibited collagenase by allowing the free carboxyl group in gallic acid to bind to the zinc site within the enzyme’s catalytic domain, potentially providing an inhibition mechanism [55]. This study highlights *R. damascena* petal extract as a potent anti-elastase inhibitor, demonstrating comparable activity to L-ascorbic acid and corilagin, both of which are known for their anti-elastase properties. These findings are supported by previous studies, which demonstrate that L-ascorbic acid inhibits elastase through the interaction of its hydroxyl groups (specifically at positions 2 and/or 3) with the histidine-57 residue of the enzyme [56]. This binding interferes with the enzyme’s active site, thereby preventing elastase from effectively hydrolyzing elastin and inhibiting its activity. On the other hand, the exact mechanism by which corilagin inhibited elastase has not been fully established. However, it may be attributed to its ability to provide abundant hydrogen bond donors (-OH groups) and receptors (oxygen atoms in both -OH and -COOR groups), allowing it to form strong interactions through multiple hydrogen bonds with the active site of elastase [57]. Notably, no single compound demonstrated significant anti-hyaluronidase activity, indicating that the observed effects are likely due to the synergistic interactions of multiple components.

### 3.6. Effect of pH on Physico-Chemical and Biological Stability of R. damascena Petal Extract

**Figure 4:**
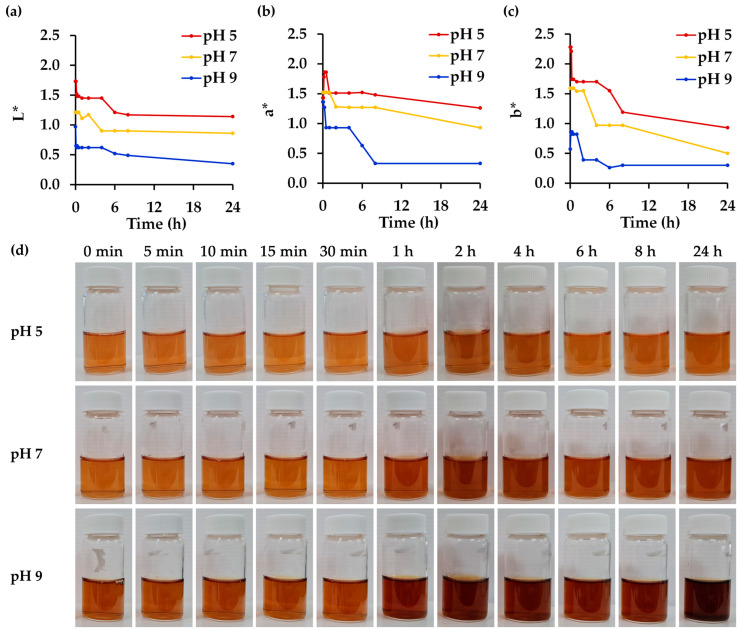
A color evaluation of *R. damascena* petal extract stored at different pH levels (pH 5, 7, and 9) under light exposure at room temperature (30 °C) for up to 24 h in terms of (**a**) lightness (L*), (**b**) green to red (a*), and (**c**) blue to yellow (b*), along with (**d**) the visual appearance of *R. damascena* petal extract. L* indicates the brightness of the sample, with higher L* values representing lighter coloration. a* represents the red–green component, with positive values indicating a shift toward red and negative values indicating green tones. b* represents the yellow–blue component, with positive values indicating increased yellow intensity, while negative values represent blue tones.

In the current study, *R. damascena* petal extract obtained through green extraction methods, particularly MAE, was found to be a highly effective active ingredient for the cosmeceutical industry, demonstrating excellent antioxidant, whitening, and anti-skin-aging activities. The cosmeceutical properties of the extract were significantly greater than those of individual chemical components widely known for their effectiveness in cosmetics. However, the stability of *R. damascena* petal extract could be a concern as anthocyanins, which are responsible for the color of the extract [58], are sensitive to environmental conditions such as pH, temperature, and light exposure. The variation in color could be a limitation when using extracts containing natural pigments, particularly in the cosmetic industry. Pigments generally absorb light in the visible range of the electromagnetic spectrum, with their color being attributed to a specific molecular group, the chromophore. However, the use of natural extracts with anthocyanins is often restricted due to color changes caused by variations in pH [59]. The effect of pH on the physical stability of *R. damascena* petal extract is shown in Figure 4. The highest pH of 9 resulted in the darkest color, with the lowest L* value and reduced red (a*) and yellow (b*) hues compared to the lower pH values of 7 and 5, respectively. A distinct change in the color of the extract at all pH values was also found over time. Upon visual inspection, extracts at pH 5 exhibited smaller changes, while more obvious changes were observed at higher pH levels, especially at pH 9. The findings are consistent with the color measurements in terms of L, a, and b* values, with a decrease in each color parameter being seen over time. Although long-term stability assessments would be valuable, the stability of the *R. damascena* petal extract in the current study was evaluated over a 24 h period. As the *R. damascena* petal extract exhibited rapid degradation, with noticeable changes occurring as early as the first hour and appearing to plateau after approximately 8 h, this early phase was considered critical and was therefore selected for in-depth evaluation at 5, 10, 15, 30, and 60 min to determine the extract’s instability. These findings are consistent with those of Bayram et al. (2015), who reported that the half-life (t_½_) of anthocyanin-rich extracts from *R. damascena* decreased shortly, with t_½_ values of 12.5 h, 3.1 h, and 1.4 h at 70 °C, 80 °C, and 90 °C, respectively, in a pH 3.5 buffer solution [60].

In addition to the observed changes in color, a reduction in the concentration of the bioactive constituents was also detected, as presented in Table 5. Rutin was noted as the most stable compound in the *R. damascena* petal extract. It remained stable at pH 7 and 9 but showed some degradation at pH 5. This is consistent with a previous study that noted rutin as more susceptible to degradation under acidic conditions, suggesting that its stability is influenced by pH [61]. Similarly, corilagin remained stable at pH 9, but some degradation was observed at lower pH levels. While most compounds tended to be more stable at a higher pH, the cyanidin-3,5-O-diglucoside concentration was found to dramatically decline at a high pH. Remarkably, the findings of this research suggest that utilizing anthocyanin levels in *R. damascena* petal extract as a parameter for stability assessment, based on their correlation with color, may potentially misrepresent the true stability of the extract. However, the cyanidin-3,5-O-diglucoside content was closely related to the color of the *R. damascena* petal extract, with the most noticeable change detected at pH 9. This is consistent with a previous study revealing that 3-glucosidic substitution strongly increased the molar absorptivity of the aglycone moiety, which favored color intensity in the alkaline region, whereas improved stability was observed at lower pH values of 2.0 and 4.0 [62]. In addition to the influence of pH on physico-chemical properties, the biological activities of the *R. damascena* petal extract were also affected (Table 5), particularly its antioxidant and anti-tyrosinase activities. In contrast, the ability of the *R. damascena* petal extract to inhibit enzymes (such as collagenase, elastase, or hyaluronidase) responsible for degrading skin structure does not depend on pH. The likely explanation for these observations is that certain key antioxidant compounds undergo chemical degradation under specific pH conditions. Ascorbic acid and gallic acid, recognized as being among the most potent antioxidants, are particularly susceptible to degradation at a low pH, which likely contributed to the diminished radical scavenging activity observed under acidic conditions. However, this reduction in activity is possibly partially offset by the synergistic interactions among the various compounds present in the *R. damascena* petal extract.

On the other hand, the radical scavenging activities for both DPPH^•^ and ABTS^•+^ were found to be reduced at a lower pH. This could be explained by the Nernst equation, which notes that an increase in pH (i.e., a decrease in hydrogen ion concentration) leads to a more negative reduction potential, enhancing the electron-donating ability of the compounds [63]. This shift would be favorable for antioxidant activity, as a lower (more negative) redox potential corresponds to a higher capacity to donate electrons and neutralize free radicals, i.e., radical scavenging. In contrast, the reducing power of the extract was found to be lower at a high pH, suggesting that the efficiency of different antioxidant mechanisms may vary depending on the pH environment.

Additionally, the observed decrease in anti-tyrosinase activity appeared to be associated with the degradation of L-ascorbic acid, which exhibits high potency, along with cyanidin-3,5-O-diglucoside and gallic acid. Therefore, the degradation of these compounds likely diminished the overall anti-tyrosinase efficacy of the *R. damascena* petal extract. On the other hand, although the pH-induced degradation reduced the concentration of some potent bioactive components of the *R. damascena* petal extract, the anti-collagenase, anti-elastase, and anti-hyaluronidase activities persisted. A possible explanation could be that the complex and overlapping contributions of multiple bioactive compounds acted in synergy.

The current study showed an effect of pH on both the physico-chemical and biological properties of *R. damascena* petal extract. Although the anti-skin-aging effects persist, chemical degradation and the resulting alterations in physical appearance are a challenge in further applications in the cosmetic industry. As the physiological pH of the skin presents an opportunity for the development of more effective topical formulations aimed at maintaining skin health, it is widely accepted that such formulations should be acidified, typically within the pH range of 4 to 6, to align with the natural acidity of the stratum corneum [64,65]. Accordingly, *R. damascena* petal extract, which demonstrated greater physical stability at pH 5, was considered advantageous for incorporation into cosmetic products. Nevertheless, although its visual appearance at this pH remained more stable compared to higher pH conditions, a significant degree of chemical and biological instability was still evident. Therefore, additional formulation strategies are required to further enhance the chemical stability of the extract and ensure its efficacy throughout the product’s shelf life. Various protective strategies have been developed to enhance the stability of bioactive constituents against environmental factors, including pH fluctuations, with particular emphasis on the use of bio-based materials such as pectin, proteins, lipids, and polysaccharides for encapsulation and controlled delivery [66]. These compounds served as effective wall materials in encapsulation systems, forming physical barriers that protect bioactive compounds, including anthocyanins, from environmental stressors [66]. Additionally, techniques such as microencapsulation, nanoencapsulation, spray-drying, freeze-drying, and liposome formation have been widely employed to improve the physico-chemical stability and bioavailability of anthocyanins [67]. Furthermore, advanced delivery systems, such as nanostructured lipid carriers (NLCs), solid lipid nanoparticles (SLNs), and emulsions, offer enhanced protection [68], making them promising strategies for preserving the functional properties of *R. damascena* petal extract in various applications, including cosmetics and cosmeceuticals.

### 3.7. Effect of Temperature on Physico-Chemical and Biological Stability of R. damascena Petal Extract

**Figure 5:**
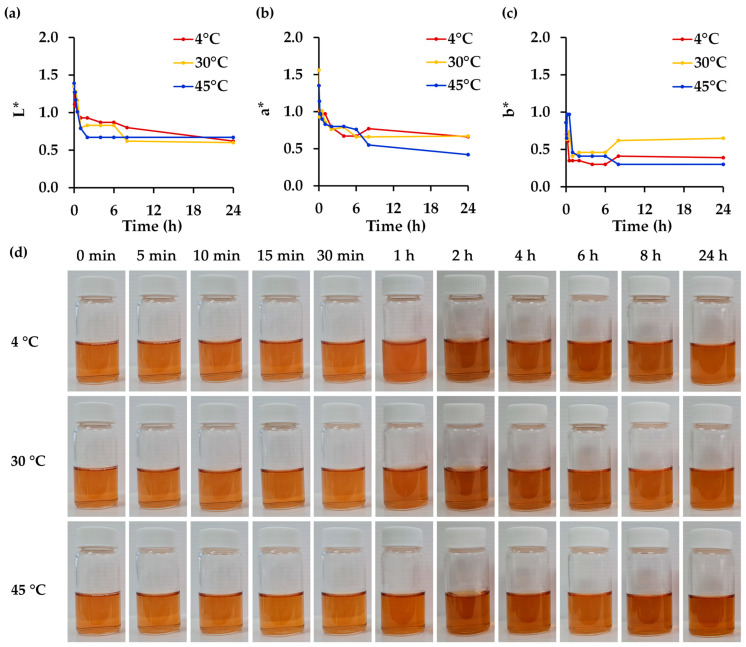
A color evaluation of *R. damascena* petal extract stored at different temperatures (4, 30, 45 °C) under light exposure at pH 5 for up to 24 h in terms of (**a**) lightness (L*), (**b**) green to red (a*), and (**c**) blue to yellow (b*), along with (**d**) the visual appearance of the *R. damascena* petal extract. L* indicates the brightness of the sample, with higher L* values representing lighter coloration. a* represents the red–green component, with positive values indicating a shift toward red and negative values indicating green tones. b* represents the yellow–blue component, with positive values indicating increased yellow intensity, while negative values represent blue tones.

The effect of temperature on the physical stability of *R. damascena* petal extract is shown in Figure 5. The *R. damascena* petal extract was stored at 4, 30, and 45 °C for 24 h, with these temperature conditions selected in alignment with internationally accepted guidelines and practical considerations for cosmetic product stability assessment [65,69,70]. This approach would reflect practical storage conditions, especially for cosmetic products designed for hot and humid climates [69]. The 4 °C condition is commonly used as a control, as low temperatures help minimize chemical and physical changes, thereby serving as a reliable baseline [70]. The 30 °C condition reflects the standard long-term storage requirement for products intended for hot and humid climates, such as those found in Southeast Asia, which falls under Climatic Zone IV (IVA and IVB), as recognized in regulatory World Health Organization (WHO) Guidance and the International Council for Harmonisation of Technical Requirements for Pharmaceuticals for Human Use (ICH) Guideline [69,71]. Finally, 45 °C is widely accepted as the upper limit for accelerated stability studies [70]. Although higher temperatures such as 50 °C, 60 °C, or 70 °C might speed up degradation, such conditions are not recommended for standard testing, as outlined by the International Federation of the Societies of Cosmetic Chemists (IFSCC), due to their potential to produce unrealistic or non-representative results [70]. Therefore, the selected temperatures provide a balanced and scientifically sound approach to assess the stability and shelf life of cosmetic products under relevant and justifiable conditions. While visual inspection did not reveal significant color changes until after 1 h of storage, quantitative color measurements detected some alterations. However, no significant differences were observed among the various temperature conditions.

In contrast to its physical appearance, temperature had significant effects on the chemical constituents of the *R. damascena* petal extract. L-ascorbic acid, gallic acid, and cyanidin-3,5-O-diglucoside were found to degrade significantly even at low temperatures, with the extent of degradation increasing markedly upon temperature increase, except in the case of gallic acid, which remained unaffected by rising temperatures. These findings are consistent with a previous study on the thermal degradation of ascorbic acid, which found that higher temperatures and longer exposure times resulted in significant losses of this compound [72]. Similarly, anthocyanins like cyanidin-3,5-O-diglucoside are sensitive to heat and can degrade rapidly at elevated temperatures, leading to a loss of color and antioxidant properties [73]. In contrast, gallic acid has been found to be more stable under thermal conditions and maintained its stability across various temperature ranges [74]. Similarly, the present study indicated chemical stability of corilagin and rutin under increasing temperatures. The findings are consistent with previous research reporting that rutin, a glycosylated flavonoid, demonstrated significant heat stability, suggesting that glycosylated flavonoids are more resistant to thermal degradation compared to their aglycone counterparts [75]. In contrast, ellagic acid exhibited greater susceptibility to thermal degradation, with a significant decrease from 106.6 ± 2.2% in storage at 4 °C to 64.8 ± 2.9% at 30 °C and 28.5 ± 9.5% at 45 °C, respectively, indicating that prolonged exposure to elevated temperatures in acidic conditions can induce the hydrolysis of ellagic acid, resulting in the formation of gallic acid and other degradation products [76].

Although storage temperatures significantly affected the bioactive content of *R. damascena* petal extract, only slight reductions in cosmeceutical efficacy were observed. The antioxidant and anti-skin-aging properties remained largely unaffected by storage temperature (Table 6), while a decline in anti-tyrosinase activity was noted, particularly when L-DOPA was used as the substrate. Additionally, rising temperatures significantly reduced antioxidant activities by decreasing the reducing ability and diminishing the anti-elastase inhibition of the extract. The reduction in the L-ascorbic acid content at elevated temperatures was identified as the primary factor contributing to the decreased reducing power, antioxidant activities, and anti-elastase effects. L-ascorbic acid has been reported as the major compound responsible for these biological effects in *R. damascena* petal extract, reinforcing its pivotal role. These findings highlight the importance of maintaining appropriate storage conditions, particularly avoiding high temperatures, to preserve the cosmeceutical properties of the extract.

### 3.8. Effect of Light Exposure on Physico-Chemical and Biological Stability of R. damascena Petal Extract

**Figure 6:**
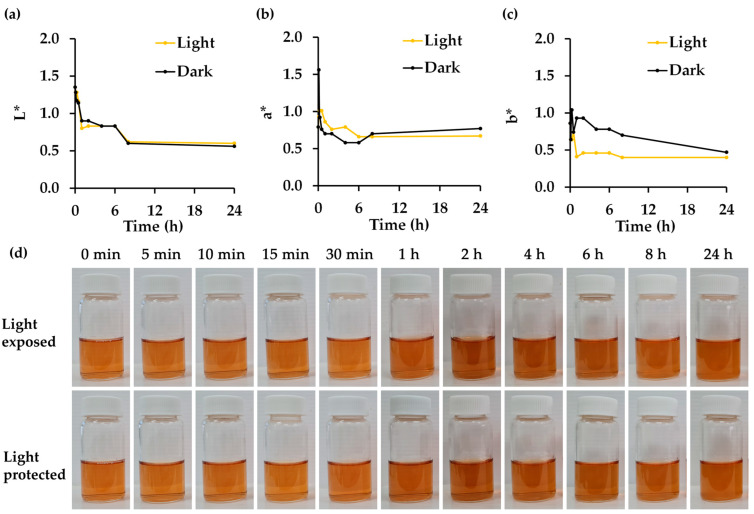
A color evaluation of *R. damascena* petal extract stored under light-exposed or light-protected conditions at room temperature (30 °C) and pH 5 for up to 24 h in terms of (**a**) lightness (L*), (**b**) green to red (a*), and (**c**) blue to yellow (b*), along with (**d**) the visual appearance of *R. damascena* petal extract. L* indicates the brightness of the sample, with higher L* values representing lighter coloration. a* represents the red–green component, with positive values indicating a shift toward red and negative values indicating green tones. b* represents the yellow–blue component, with positive values indicating increased yellow intensity, while negative values represent blue tones.

The effect of light exposure on the physical stability of *R. damascena* petal extract is shown in Figure 6. Visual inspection did not reveal significant color changes over the storage duration or between the light-exposed and light-protected conditions. The results are in line with a previous study, which revealed that no significant color change was observed regardless of exposure to light or darkness [77]. However, quantitative color measurements could detect alterations, with the b* value decreasing in the light-exposed extract.

Aside from the impact on the physical stability of *R. damascena* petal extract, light exposure significantly affected nearly all of its chemical constituents, except for cyanidin-3,5-O-diglucoside and corilagin (Table 7). The findings align with previous studies, which reported a 5% reduction in the anthocyanin content following light exposure [77]. In contrast, L-ascorbic acid exhibited significant degradation under light exposure. It has been documented that L-ascorbic acid is highly susceptible to light, undergoing oxidative degradation to dehydroascorbic acid, which subsequently further degraded into inactive products [78,79]. Similarly, gallic acid has been shown to degrade when exposed to UV light, with approximately 50% degradation occurring after 3 h of UV-C exposure [74]. Rutin and ellagic acid have been reported to degrade upon exposure to UV light [80,81].

Regarding the cosmeceutical properties, reductions were observed only in DPPH^•^ inhibition, anti-tyrosinase, and anti-elastase activities. This could likely be attributed to the significant decrease in the L-ascorbic acid content, which was identified as a key contributor to these biological activities. The findings from this study suggest the use of light-protected containers for *R. damascena* petal extract to preserve its physico-chemical and biological properties.

## 4. Conclusions

This study presented a novel sustainable extraction method for *R. damascena* using MAE, yielding a highly concentrated, multifunctional botanical active without the use of organic solvents. The extract exhibited substantial potential as a valuable active ingredient in the cosmeceutical industry, demonstrating antioxidant, whitening, and anti-skin-aging effects. Notably, the cosmeceutical properties of the extract were found to be more potent than those of individual chemical compounds commonly used in cosmetics, including L-ascorbic acid, gallic acid, cyanidin-3,5-O-diglucoside, corilagin, rutin, and ellagic acid. However, the stability of *R. damascena* petal extract was identified as a potential concern, influenced by environmental factors, including pH, temperature, and light exposure. pH mainly affected the physical appearance of the *R. damascena* petal extract, while temperatures and light exposure mainly affected its chemical content. Anthocyanin can be suggested as a marker for the physical stability of *R. damascena* petal extract, but not for its biological stability. L-ascorbic acid, which is responsible for antioxidant, anti-tyrosinase, and anti-elastase properties, was identified as the primary factor contributing to the decline in these cosmeceutical activities upon exposure to varying pH levels, elevated temperatures, and light. Corilagin, the most abundant compound in *R. damascena* petal extract, was found to be stable under different pH levels, temperature conditions, and light exposure. The findings from this research underline the importance of proper storage conditions for preserving the cosmeceutical efficacy of *R. damascena* petal extract. It is recommended to avoid high temperatures and light exposure for *R. damascena* petal extract to preserve its stability and efficacy. A low pH of 5 adversely affected the chemical constituents and cosmeceutical activities of the extract, which should be considered as a potential concern for future skincare product development. To facilitate the integration of *R. damascena* petal extract into cosmetic products, strategies to enhance its stability and efficacy are recommended, including the use of advanced delivery systems such as NLCs, SLNs, and liposomes. These systems not only protect the extract’s bioactive compounds from degradation caused by pH variation, temperature fluctuations, and light exposure, but also enhance skin penetration and extend shelf life, supporting its application in anti-aging formulations.

## Figures and Tables

**Table 1:** Chemical compositions of *R. damascena* petal extracts obtained using green extraction methods.

Compounds	RT (min)	Chemical Content (mg Per g *R. damascena* Petal Extract)					
		INF	UAE	MCE-T20	MCE-T80	MAE	PEF
L-ascorbic acid	2.60	3.59 ± 0.00 ^e^	3.97 ± 0.00 ^d^	5.50 ± 0.01 ^c^	5.62 ± 0.01 ^b^	9.97 ± 0.01 ^a^	3.98 ± 0.00 ^d^
Gallic acid	3.62	7.05 ± 0.00 ^d^	8.20 ± 0.01 ^c^	8.77 ± 0.01 ^b^	8.75 ± 0.01 ^b^	72.56 ± 0.03 ^a^	6.41 ± 0.01 ^e^
Cyanidin-3,5-O-diglucoside	5.24	14.11 ± 0.01 ^c^	14.29 ± 0.01 ^c^	0.00 ± 0.00 ^d^	0.00 ± 0.00 ^d^	82.92 ± 0.02 ^a^	15.30 ± 0.01 ^b^
Corilagin	8.46	34.30 ± 0.01 ^c^	34.90 ± 0.02 ^b^	23.29 ± 0.01 ^e^	14.68 ± 0.01 ^f^	213.59 ± 0.01 ^a^	25.16 ± 0.01 ^d^
Rutin	13.21	4.30 ± 0.01 ^d^	3.81 ± 0.01 ^e^	4.74 ± 0.01 ^c^	5.21 ± 0.01 ^b^	6.70 ± 0.01 ^a^	3.62 ± 0.00 ^f^
Ellagic acid	14.74	22.97 ± 0.01 ^d^	22.37 ± 0.01 ^e^	23.74 ± 0.01 ^c^	24.67 ± 0.01 ^b^	48.49 ± 0.01 ^a^	17.45 ± 0.01 ^f^

NOTE: *R. damascena* petals were extracted using various green extraction methods, including infusion (INF), ultrasonic-assisted extraction (UAE), micellar extraction using Tween^®^ 20 (MCE-T20), micellar extraction using Tween^®^ 80 (MCE-T80), microwave-assisted extraction (MAE), and pulsed electric field extraction (PEF). Different letters (a–f) denote significant differences in the content of each chemical composition among various R. damascena petal extracts, analyzed using a one-way ANOVA, followed by Tukey’s post hoc test (*p* < 0.05).

**Table 2:** The antioxidant activities of *R. damascena* petal extract.

Samples	DPPH Inhibition IC_50_ (µg/mL)	TEAC (mg Trolox/g)	EC_1_ (mM FeSO_4_/g)
L-ascorbic acid	4.8 ± 0.2 ^c^	17.0 ± 0.3 ^b^	13.4 ± 0.6 ^b^
Gallic acid	1.0 ± 0.1 ^b^	14.2 ± 1.8 ^c^	14.2 ± 1.8 ^b^
Cyanidin-3,5-O-diglucoside	33.4 ± 8.2 ^d^	1.9 ± 0.2 ^d^	0.7 ± 0.1 ^d^
Corilagin	31.4 ± 0.2 ^d^	16.7 ± 0.1 ^b^	5.0 ± 0.1 ^c^
Rutin	43.8 ± 5.1 ^e^	19.4 ± 0.2 ^a^	5.6 ± 0.0 ^c^
Ellagic acid	4.8 ± 0.2 ^c^	3.9 ± 0.2 ^d^	14.7 ± 0.6 ^b^
*R. damascena* petal extract	0.5 ± 0.1 ^a^	21.2 ± 0.3 ^a^	53.1 ± 1.7 ^a^

NOTE: *R. damascena* petal extract was obtained using microwave-assisted extraction for 3 min (MAE). Different letters (a–e) denote significant differences in antioxidant activities among various samples, analyzed by one-way ANOVA, followed by Tukey’s post hoc test (*p* < 0.05).

**Table 3:** The anti-tyrosinase activities of *R. damascena* petal extract.

Samples	Anti-Tyrosinase Activity (%)	
	Tyrosine	L-Dopa
Kojic acid	93.1 ± 0.2 ^a^	96.2 ± 0.9 ^a^
L-ascorbic acid	44.1 ± 4.9 ^b^	42.6 ± 1.8 ^b^
Gallic acid	19.3 ± 1.7 ^d^	29.7 ± 1.0 ^c^
Cyanidin-3,5-O-diglucoside	32.3 ± 1.6 ^c^	18.9 ± 1.5 ^d^
Corilagin	20.6 ± 1.3 ^d^	28.0 ± 5.7 ^c^
Rutin	6.3 ± 1.7 ^e^	0.7 ± 4.1 ^e^
Ellagic acid	19.0 ± 0.3 ^d^	17.9 ± 1.0 ^d^
*R. damascena* extract	93.9 ± 1.8 ^a^	95.6 ± 0.0 ^a^

NOTE: *R. damascena* petal extract was obtained using microwave-assisted extraction (MAE) for 3 min. Different letters (a–e) denote significant differences in the anti-tyrosinase activities among various samples, analyzed by one-way ANOVA, followed by Tukey’s post hoc test (*p* < 0.05).

**Table 4:** The anti-aging activities of *R. damascena* petal extract.

Samples	Anti-Collagenase Activity (%)	Anti-Elastase Activity (%)	Anti-Hyaluronidase Activity (%)
EGCG	74.7 ± 2.6 ^a^	46.0 ± 8.2 ^c^	80.6 ± 1.0 ^b^
Oleanolic acid	81.6 ± 0.8 ^a^	71.8 ± 0.5 ^a^	78.4 ± 2.0 ^b^
L-ascorbic acid	54.4 ± 2.0 ^c^	67.5 ± 10.6 ^a,b^	7.6 ± 3.6 ^d^
Gallic acid	66.4 ± 1.4 ^b^	68.4 ± 8.3 ^b^	0.9 ± 4.5 ^d^
Cyanidin-3,5-O-diglucoside	10.6 ± 0.9 ^d^	0.0 ± 1.4 ^d^	17.0 ± 5.3 ^c^
Corilagin	16.7 ± 0.5 ^d^	61.1 ± 2.5 ^a,b,c^	8.3 ± 2.8 ^c,d^
Rutin	10.8 ± 5.1 ^d^	0.0 ± 4.1 ^d^	1.6 ± 1.8 ^d^
Ellagic acid	0.0 ± 5.8 ^e^	0.0 ± 9.3 ^d^	19.6 ± 3.0 ^c^
*R. damascena* extract	69.5 ± 0.8 ^a^	75.3 ± 4.5 ^a,b^	89.6 ± 0.5 ^a^

NOTE: *R. damascena* petal extract was obtained using microwave-assisted extraction (MAE) for 3 min. EGCG = epigallocatechin gallate. Different letters (a–e) denote significant differences in the anti-skin aging activities among various samples, analyzed by one-way ANOVA, followed by Tukey’s post hoc test (*p* < 0.05).

**Table 5:** Percentage of remaining chemical constituents and biological activities of *R. damascena* petal extract after storage at various pH levels, at room temperature (30 °C), with light exposure for 24 h.

Chemical and Biological Properties	pH		
	5	7	9
Chemical constituents			
L-ascorbic acid	31.4 ± 1.4 ^c^	50.4 ± 6.9 ^b^	99.9 ± 2.5 ^a^
Gallic acid	50.1 ± 1.8 ^b^	52.4 ± 1.3 ^b^	81.0 ± 2.0 ^a^
Cyanidin-3,5-O-diglucoside	52.2 ± 2.5 ^a^	53.0 ± 0.9 ^a^	22.4 ± 2.7 ^b^
Corilagin	83.6 ± 1.2 ^b^	86.4 ± 1.9 ^b^	110.3 ± 4.9 ^a^
Rutin	91.1 ± 1.3 ^b^	104.0 ± 1.8 ^a^	103.8 ± 1.1 ^a^
Ellagic acid	64.8 ± 2.9 ^b^	74.9 ± 3.1 ^a^	74.4 ± 0.2 ^a^
Biological activities			
DPPH^•^ inhibition	68.1 ± 9.2 ^b^	83.9 ± 4.9 ^a,b^	96.5 ± 3.7 ^a^
ABTS^•+^ inhibition	99.6 ± 0.2 ^b^	104.3 ± 0.6 ^a^	103.6 ± 0.2 ^a^
Reducing power	102.7 ± 1.0 ^b^	112.7 ± 1.0 ^a^	87.2 ± 0.3 ^c^
Anti-tyrosinase (L-tyrosine)	70.6 ± 5.6 ^b^	69.1 ± 4.6 ^b^	91.6 ± 8.4 ^a^
Anti-tyrosinase (L-DOPA)	72.7 ± 6.2	60.9 ± 2.3	70.4 ± 6.2
Collagenase inhibition	95.8 ± 3.1	99.5 ± 1.9	101.3 ± 3.0
Elastase inhibition	93.9 ± 2.7	94.3 ± 4.6	100.1 ± 3.2
Hyaluronidase inhibition	89.7 ± 5.6	87.8 ± 9.8	82.9 ± 9.7

NOTE: EGCG = epigallocatechin gallate. Different letters (a–c) denote significant differences in the remaining chemical content or biological activities among the samples stored at different pH levels for 24 h, analyzed by one-way ANOVA, followed by Tukey’s post hoc test (*p* < 0.05).

**Table 6:** Percentage of remaining chemical constituents and biological activities of *R. damascena* petal extract after storage at various temperatures, at pH 5, with light exposure for 24 h.

Chemical and Biological Properties	Temperature (°C)		
	4	30	45
Chemical constituents			
L-ascorbic acid	60.4 ± 5.9 ^a^	31.4 ± 1.4 ^b^	28.1 ± 3.8 ^b^
Gallic acid	46.4 ± 2.0	50.1 ± 1.8	47.3 ± 0.2
Cyanidin-3,5-O-diglucoside	61.3 ± 3.6 ^a^	52.2 ± 2.5 ^a,b^	50.9 ± 5.2 ^b^
Corilagin	87.7 ± 2.2	83.6 ± 1.2	85.0 ± 5.2
Rutin	90.7 ± 2.1	91.1 ± 1.3	88.6 ± 1.6
Ellagic acid	106.6 ± 2.2 ^a^	64.8 ± 2.9 ^b^	28.5 ± 9.5 ^c^
Biological activities			
DPPH^•^ inhibition	82.3 ± 5.7	68.1 ± 9.2	68.1 ± 9.2
ABTS^•+^ inhibition	99.4 ± 0.2	99.6 ± 0.2	99.5 ± 0.1
Reducing power	100.9 ± 0.3 ^b^	102.7 ± 1.0 ^a^	97.1 ± 0.6 ^c^
Anti-tyrosinase (L-tyrosine)	74.7 ± 3.1	70.6 ± 5.6	68.6 ± 5.7
Anti-tyrosinase (L-DOPA)	85.8 ± 7.3 ^a^	72.7 ± 6.2 ^a,b^	63.8 ± 8.6 ^b^
Collagenase inhibition	98.9 ± 2.4	95.8 ± 3.1	95.2 ± 3.5
Elastase inhibition	91.9 ± 0.7 ^a^	93.9 ± 2.7 ^a^	77.9 ± 5.0 ^b^
Hyaluronidase inhibition	92.2 ± 6.0	89.7 ± 5.6	87.6 ± 6.5

NOTE: EGCG = epigallocatechin gallate. Different letters (a–c) denote significant differences in the remaining chemical content or biological activities among the samples stored at different temperatures, at pH levels of 5, analyzed by one-way ANOVA, followed by Tukey’s post hoc test (*p* < 0.05).

**Table 7:** Percentage of remaining chemical constituents and biological activities of *R. damascena* petal extract after storage at room temperature (30 °C), at pH 5, under light-exposed or light-protected conditions for 24 h.

Chemical and Biological Properties	Condition	
	Light Exposure (Light)	Light-Protected (Dark)
Chemical constituents		
L-ascorbic acid	31.4 ± 1.4	58.9 ± 1.9 ***
Gallic acid	50.1 ± 1.8	56.1 ± 1.8 *
Cyanidin-3,5-O-diglucoside	52.2 ± 2.5	51.3 ± 5.2
Corilagin	83.6 ± 1.2	81.5 ± 2.7
Rutin	91.1 ± 1.3	96.7 ± 1.9 *
Ellagic acid	64.8 ± 2.9	74.3 ± 2.7 *
Biological activities		
DPPH^•^ inhibition	68.1 ± 9.2	101.4 ± 5.8 ***
ABTS^•+^ inhibition	99.6 ± 0.2	99.4 ± 0.0
Reducing power	102.7 ± 1.0	106.9 ± 0.4
Anti-tyrosinase (L-tyrosine)	70.6 ± 5.6	82.1 ± 1.9 **
Anti-tyrosinase (L-DOPA)	72.7 ± 6.2	83.4 ± 8.0
Collagenase inhibition	95.8 ± 3.1	94.9 ± 3.5
Elastase inhibition	93.9 ± 2.7	77.9 ± 5.0 *
Hyaluronidase inhibition	89.7 ± 5.6	87.3 ± 7.0

NOTE: EGCG = epigallocatechin gallate. Asterisk(s) denote significant differences in chemical content among samples under light-exposed or light-protected conditions at room temperature (30 °C), at pH 5, for 24 h, analyzed by pair sample *t*-test (* *p* < 0.05, ** *p* < 0.01, and *** *p* < 0.001).

## Data Availability

The original contributions presented in this study are included in the article; further inquiries can be directed to the corresponding author.

## Abbreviations

The following abbreviations are used in this manuscript:

AAAVPN	N-Succinyl-Ala-Ala-Ala-p-nitroanilide
ABTS	2,2-Azino-bis3-ethylbenzothiazoline-6-sulfonic acid
BSA	Bovine serum albumin
DI	Deionized
DPPH	2,2-Diphenyl-1picrylhydrazyl
EC_1_	Equivalent capacity
ECM	Extracellular matrix
EGCG	Epigallocatechin gallate
FALGPA	2-Furanacryloyl-Leu-Gly-Pro-Ala
HPLC	High performance liquid chromatography
IC_50_	Half-maximal inhibitory concentration
INF	Infusion
MAE	Microwave-assisted extraction
MCE	Micellar extraction
MCE-T20	Micellar extraction using Tween^®^ 20
MCE-T80	Micellar extraction using Tween^®^ 80
MMP-1	Metalloproteinase-1
NLCs	Nanostructured lipid carriers
PEF	Pulsed electric fields extraction
SLNs	Solid lipid nanoparticles
TEAC	Trolox equivalent antioxidant capacity
TPTZ	2,4,6 Tripyridyl-s-triazine
UAE	Ultrasound-assisted extraction
UV	Ultraviolet
